# Fluorescent lamp tungsten filament thermionic emission gun as a novel humidity optical sensor

**DOI:** 10.1038/s41598-021-97688-4

**Published:** 2021-09-13

**Authors:** Hossein Torabi-Monfared, Leila Sherafat, Mohammad Mahdi Doroodmand, Fazlolah Eshghi

**Affiliations:** Department of Chemistry, College of Sciences, Shiraz University, Shiraz, Iran

**Keywords:** Optical sensors, Optics and photonics, Optical techniques, Imaging and sensing

## Abstract

Detecting humidity have been remained a continuing concern within some important areas such as structural health, food processing, industrial as well as agricultural products. In this study, a novel humidity optical sensor is introduced based on the thermionic emission of tungsten filament using the fluorescent lamp set-up. Estimated blue compliant using a charged coupling device camera in optical image of the tungsten filament was confirmed as an appropriate detection system for relative humidity (RH) sensing. The fabricated optical sensor has wide linear range (2.0–98% RH), improved detection limit (< 5.0% RH), acceptable saturated limit (> 99.0% RH), improved percentage of relative standard deviation (4.18%, n = 2), adequate hysteresis (< 4.0% RH) and a shorter rise time (< 5.0 s), respectively. The mechanism behind this detection system is based on the interaction between H_2_O and tungsten filament during formation of W${\mathrm{O}}_{3}$.x ${\mathrm{H}}_{2}$O (x = 1–2) in terms of some spectroscopic obtained evidences as well as Fourier transform infrared and X-ray diffraction spectrometries.

## Introduction

For many years, how to measure and monitor the relative humidity (RH) amount has been a matter of debate in different industries, agriculture, and social health^1–3^. However, the wide range of monitoring humidity sensing shows strong interest in developing novel RH sensors. Most reported humidity sensors using two main electrical (resistive and capacitive) and optical properties of proposed materials can be classified in two groups: chemical reaction and physical interaction^4,5^. Chemical reaction based-sensors comprise of a transducer and an active layer to convert the chemical information into another form of electrical signal gradients like frequency, current, voltage, etc.^6–8^. Their performance characteristic is often based on some different figures of merit including sensitivity, selectivity, detection limit, response time, recovery time, and so on). That’s why, many research groups have approached to the promotion of humidity-sensitive materials and the current existing manufacturing process^9,10^, especially, SnO_2_^11^, SiO_2_^12^, carbon allotropes^13^, zeolites^14^, nanohybrid^15^, tungsten based materials ( W, WO_3_, WS_2_ and their hybrids with other compounds)^16–19^, etc.^20,21^.

Among the introduced sensing probes, tungsten based sensors are strongly attracted from the scientists all over the world. For instance, based on the literature, WO_3_, nanoparticles decorated WS_2_ hetero-junction have been adopted for the highly sensitive ethanol gas sensing application^16^. In addition, layer-by-layered self-assembled WS_2_ has been incorporated with other compounds like TiO_2_ as nanocomposite for the humidity sensing performance^17^. Also, other tungsten based compounds such as tungsten(VI) oxide, modified with the molybdenum(VI) oxide has been selected for evaluation of the electrical and humidity sensing properties^18^.

About most of these gas sensing devices, in spite of their capability such as their moderated selectivity for gas sensing purposes^16–18^, but they often suffer from problems such as small sensitivity, high hysteresis, fatigue and or lack of reusability. However, to solve these problems, it has been focused on the nanotechnology such as W nanowires^19^, but, regardless of noticeable advantages of chemical reaction based-sensors, they are influenced by some drawbacks such as small water permeability, irreversible water adsorption/absorption resulting in significant memory effects, besides the inadequate hysteresis), and low selectivity against H_2_O compared to other organic/inorganic species, especially volatile organic compounds^22,23^. Although, doping different nanostructures to the existing sensors have partially promoted the active surface area, and accessed acceptable permeability and selectivity^24,25^. This process often achieved via following main electrical^26,27^ and/or optical^28–30^ properties in the manufacturing processes of different RH sensors. Indeed, optical sensors are more useful than the electrical sensing devices, due to the intrinsic features like protection to electrical noises and facility of the miniaturization^28–30^. The main aim of current study is fabricating a novel humidity optical sensor via chemical reaction between water vapor and tungsten based on the thermionic emission of tungsten (*W*) filament, simply using fluorescent lamp instrumentation system (gun) with significant advantages such as low cost, availability and/or almost simple detection systems.

## Experimental

### Reagents and materials

Tungsten filament with 2.50 ± 0.03 diameter and electrical resistivity of 2.0 ± 0.1 Ω cm^−1^ was related to the Fras Tungsten Company (Shiraz, Iran). Analytical grade of KBr was purchased from Merck Company. Triply distilled water (Conductivity: 0.5 μS cm^−1^) was adopted from the combined cycle power plan (Shiraz, Iran) to make the RH standard solutions. Analytical grades of gases with different purities (weight percentages, W/W) such as nitrogen (N_2_ 99.1%), hydrogen (H_2_, 99.996%), carbon monoxide (CO, 99.992%,), carbon dioxide (CO_2_, 99%), argon (Ar, 99.992%) and helium (He, 99.9997%) were from Parsballoon Company (Shiraz, Iran). Also, different volatile organic compounds (VOCs) with analytical grades such as absolute ethanol (Merck Company, 99%, W/W), diethyl methyl ether (Merck Company, 95%, W/W), acetone (Flucka Company, 99.0%, W/W), etc. were selected. In addition, 5.0 ± 0.1 mL of mixture of exhausting gas of a vehicle (L_90_ automobile, Pars Khodro, Class: 56,080 2017, Tehran, Iran) was directly sampled inside a tygon tube (200.0 mL, Saint Gobain Fluid Transfer Tygon® F-4040-A, Kyalami Business Park, Kyalami, Midrand, South Africa) to estimate the probable interfering effect(s).

### Instruments

For sensitive and selective RH sensing process, it was focused on the thermionic radiation of tungsten filament as optical detection system. For this purpose, briefly, a new system was designed during formation of the RH standard solutions, ranged between 2.0 and 98.0% (± 0.1), using a cooling mist piezoelectric-based humidifier (Dyson Pure Humidify + Cool Ph01, 12.0 V, Direct Current, DC, China). The humidity system was situated inside a closed cylindrical plastic container (50.0 mL), half filled with the triply distilled water. Introduction of the humidity was achieved via bubbling Ar as carrier gas into the water fluid (from the inlet port of the container), at a fixed flow rate, set by a mass flow controller (MFC), (GE50A013503RMV020 Mass Flow Controller, Germany). The adequate volume of gaseous sample was then transferred from the outlet port of the container. Under this condition, the humidity standard solutions (inside Ar gas as a solvent) were directly standardized via controlling the Ar flow rate as well as the time of the operation of the humidity system. RH% using a reference RH probe (GCH-2018, ISO-9001, CE, EU) was continuously monitored.

Image processing of the system was selected as detection system using a charge coupled device (*CCD*) camera (UOP0600CS, USB 3.0 CCD Camera, Microscope Biological C Mount Microscope Camera, 6.0 Million Pixel, China).

The pressure of the system was also controlled a vacuum pump (Vacuum pump, VP280, 283.0 L min^−1^, 10.0 CFM, dual stage, Germany). In addition, the temperature of the RH standard solutions was also controlled using a tungsten carbide tubing furnace (length: 20.0 cm, e.d.: 15.0, i.d. 8.0 cm, Azar Furnace Company, Tehran, Iran), situated around the water container.

All the effective parameters such as type of the filament, conditioning the tungsten filament, length of the tungsten filament, electrical applied potential, pressure and the temperature of the system were automatically controlled using an electronic interface (PCF8591-8-Bit Analog Digital Analog Converter ADC/DAC, read relay, 5.0 V DC, analog device, China) through the RS-232 port of a PC and a program software, written in Visual Basic 6 (VB_6_) software.

The probable mechanism of the RH detection system was evaluated using spectroscopic techniques such as Fourier transform- infrared (FT-IR, Shimadzu, 8000 Seri, Japan), and X-ray diffraction pattern (XRD, AXS Bruker, US). In addition, a digital caliper (RS PRO, China) was adopted to estimate the length and diameter of the utilized modules in this system.

### Apparatus

**Figure 1:**
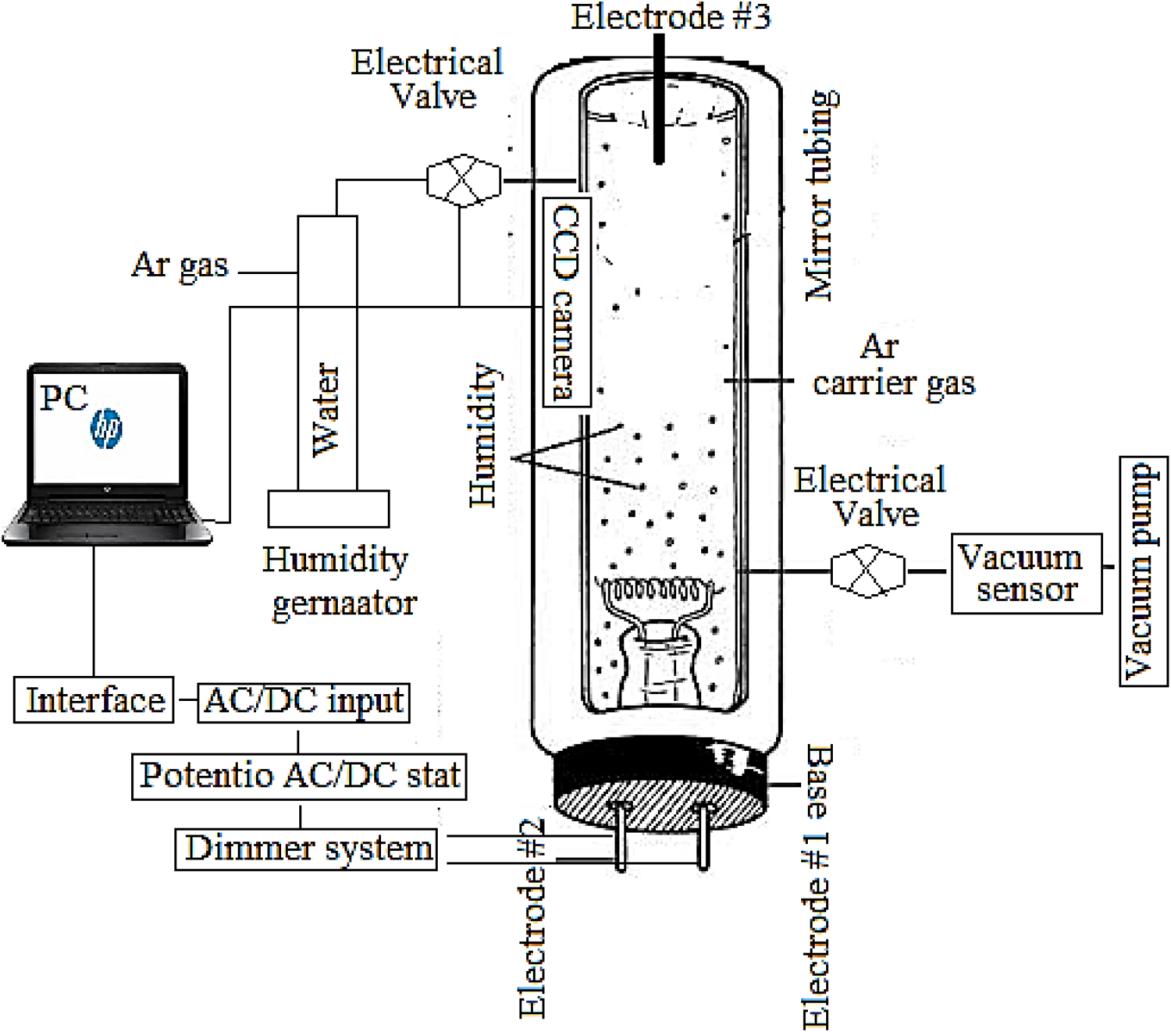
Schematic of the designed apparatus for humidity sensing process.

Figure 1 shows the schematic of the designed apparatus for the RH detection and measurement. To evaluate the capability of the tungsten filament during interaction with the water molecules, a general fluorescent lamp (Voltage: 220–240 V, AC and power: 20.0 W, KHazar Power, Tehran, Iran) was purchased. After that, the tungsten holder (i.e., electron gun) was separated via cutting the fluorescent tubing from 2.00 ± 0.01 cm higher than the position of the tungsten holder (safety measures were performed). The gas containing water vapor (humidity) was then connected to the fluorescent lamp-like set-up through a Pyrex glass tubing with 5.0 cm diameter and 1.0 cm height. Argon was also selected as carrier gas instead of air through as two-way valves (Brass/Bronze, 12.0 V DC, normally closed, Two-Way Solenoid Valve, India).

All the system was positioned inside a cylindrical mirror light pipe (MLP, 1415-RP, China) with 5.00 cm diameter and 22.00 cm height. The CCD camera was also introduced to the MLP. The applied AC (alternative current) electrical potential to the tungsten filament was also controlled using as voltage divider analog device through an analog Variac (Variable transformer, 210–240 V, AC, single phase with digital display, Pars Technique, China).

The electrical potential was applied through a three-electrode system, including two florescent electrodes (electrodes: #1 and #2), as the negative-phase pins, which were connected to the two sides (ends) of the tungsten filament inside the analyzing volume. The third electrode included a graphite rod (Electrode #3, Purity: > 99.0%, W/W, length: 2.00 ± 0.01 cm, diameter 2.00 ± 0.01 mm, Saba-Battery company, Tehran, Iran) as the pseudo reference electrode (Null pin). This electrode was connected to the ground (GND) of the electrical supply. Under this condition, the electrical current flow from µA to mA levels was correlated to the electron emission process between the tungsten filament and the reference electrode during appearance of the thermionic radiations.

In addition, the electrical current of the thermionic process was amplified through a general fluorescent lamp driver circuit (220 V AC*,* 555 Timer IC, 20.0 W, Analog Device, US). The pressure of the cell was also controlled at 25–30 torr using the vacuum pump. This was controlled using as three-way valve Brass/Bronze, 12.0 V DC, Normally closed, Three Way Solenoid Valve, India), positioned inside the MLP. The vacuum of the system was then monitored using a reference vacuum meter (Sunshine Instruments Coimbatore, Tamil Nadu). All the components including, valve, vacuum pump, vacuum meter as well as the applied voltage to the filament were controlled automatically through an electronic circuit and a computer program written in the VB_6_.

### Procedure

Before starting the analysis process, the Ar gas was purged to the analyzing volume with flow rate of 5.0 mL min^−1^ for 2.0 min to have full confidence about the lack of attendance of any oxygen molecule as the oxidant or memory effect(s) of H_2_O vapors as analyte in the system. After turning off the Ar purging, the pressure of the detection system was set to 25–30 torr using the vacuum pump, along with directly monitoring the pressure by the reference pressure sensor. Then, the AC electrical potential as 1arge as 110 ± 1 V (AC, vs. GND) was applied to the electrode system, and aged for 2.0 min to have complete assurance about the thermal stability as well as steady state condition for the electron emission process. Formerly, the humidifier (humidity system) as well as the MFC were turning on to introduce a fixed RH% standard solution to the system, together with simultaneous standardizing using the reference RH probe. This process therefore led to transfer the transient amount of the humidity from the humidity container inside the vacuum system. Afterward, the CCD was set and the photographic images were monitored vs. time. After the analysis process, the electrical power supply, the humidity and the electrical valves were sequentially switched off. The memory effect was subsequently eliminated via following the vacuum processor for 3.0 min for making the system ready for the next experiments. Summation of blue component, as a humidity detection probe, of each pixel was performed using the VB_6_ program.

### Real sample analysis

The reliability of this sensor was evaluated via analyses of different real gas samples such as urban tunnel, lab air, automobile exhaust air, etc. For this purpose, each sample was accumulated in a plastic balloon (30.0 mL) using a membrane pump (Tornado AC580, 12 V DC, 150 psi, China). After cleaning the glass cell as well as elimination of any probable memory effect(s) from the previous analysis, using the procedure reported in the previous section, each sample was introduced to the cell directly with a flow rate of 2.00 ± 0.08 mL min^−1^ for 1.0 min time interval and the obtained photographic images were processed according to the recommended procedure.

### Optimization

In this study, one-at-a time method was selected to optimize the proposed method. Optimization was based on estimating the summation of each red, green and blue (RGB) components (as well as their linear combinations such as R + G, R + B and G + B) of each pixel as the detection system. The reproducibility as well as the reparability of this system were estimated based on the calculation of the percentage of the relative standard deviation (RSD%) during at least three replicate analyses. In addition, the uncertainty of each datum was based on the estimation of the ± RSD (n > 3).

## Results and discussion

Based on literature, thermionic emission of tungsten filament is considered as one of the most important topics during formation of electron source for bombardment purposes^31^. This system also plays role as a simple and controllable thermal sources for different kinds of thermo-reactions, especially those dealing with the endothermic processes^31^. One of the reactions is the chemical interaction between tungsten and water molecules^32,33^. This interaction is often accompanied with formation of thermionic emission (radiation)^31^ whose selectivity is related to that of the reaction. About some physical processes such as thermal sources that are operated based on the electrical current flow from the filament, formation of radiation is not so selective^31^; whereas when these processes are originated from an electrochemical phenomenon, at constant physical conditions, the selectivity of this process seems to be more acceptable^33^. Based on these phenomena, hereby in this report, for the first time, the electrochemical interaction between the tungsten filament and water molecules is evaluated based on the driving force of the electron radiation. This interaction has therefore resulted in introducing a reliable optical RH sensor.

### Structure characterization

Effective interaction between tungsten filament and water molecules was evaluated via spectroscopic methods: FT-IR spectrometry and X-ray diffraction. It should be noted that, for the preparation of the FT-IR samples, the used tungsten filament was mixed enough with a dry KBr powder in the mortar for mechanically contacting with the solid powder. After that, the powder was pressed and analyzed by the FT-IR spectrometer.

**Figure 2:**
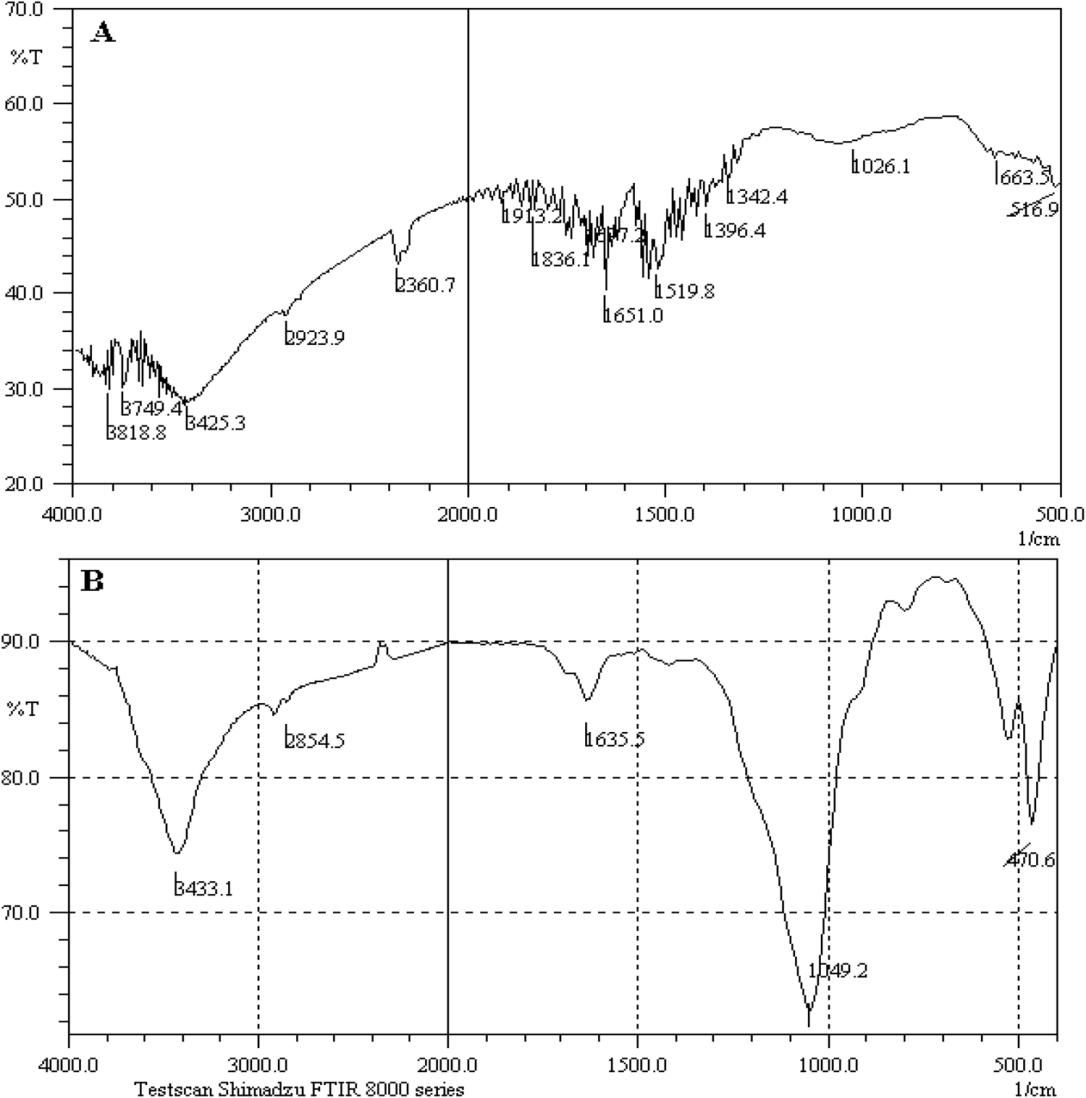
Surface analyses of the tungsten filament by FT-IR spectrometry during interacting with water vapor (**A**) before and (**B**) after applying the electrical potential. Conditions: standard RH: 40–45%, applied voltages: 110 ± 1 V (AC, vs. GND), and pressure: 25–30 torr.

However, it should be noted that, due to the small size of the modified W filament as well as its frugality, it was impossible to analyze the sample using FT-IR spectrometry, equipped with attenuated total reflectance (ATR) accessory. In addition, as it was needed to crash the used tungsten filament to perform this experiment; consequently, it was difficult to obtain FT-IR spectra with high enough resolution. In addition, probably, trace amount of the sample prevented the observation of related functional groups using near IR spectrometry. Nevertheless, the change in the functional groups of the modified W filament, before and after humidity introduction at RH > 60% was clearly evidenced according to the middle FT-IR spectra as shown in Fig. 2. All these results pointed to the formation of thin film of on the W filament.

Based on the FT-IR spectra (Fig. 2), the peaks positioned at 3440 and 1604 cm^−1^ were attributed to the adsorbed water^34^. In addition, the peaks situated at frequency of 440–475 and ~ 1050 cm^−1^ were related to the formation of O-W–O bendings^35,36^, as the product during reaction between tungsten filament and water vapor. In addition, major difference was observed between the water interacted W-based filament before (A) and after (B) applying the electrical potential to the system under the optimum conditions. Consequently, significant interaction was evidenced between tungsten and water vapor during the humidity detection and measurement process.

**Figure 3:**
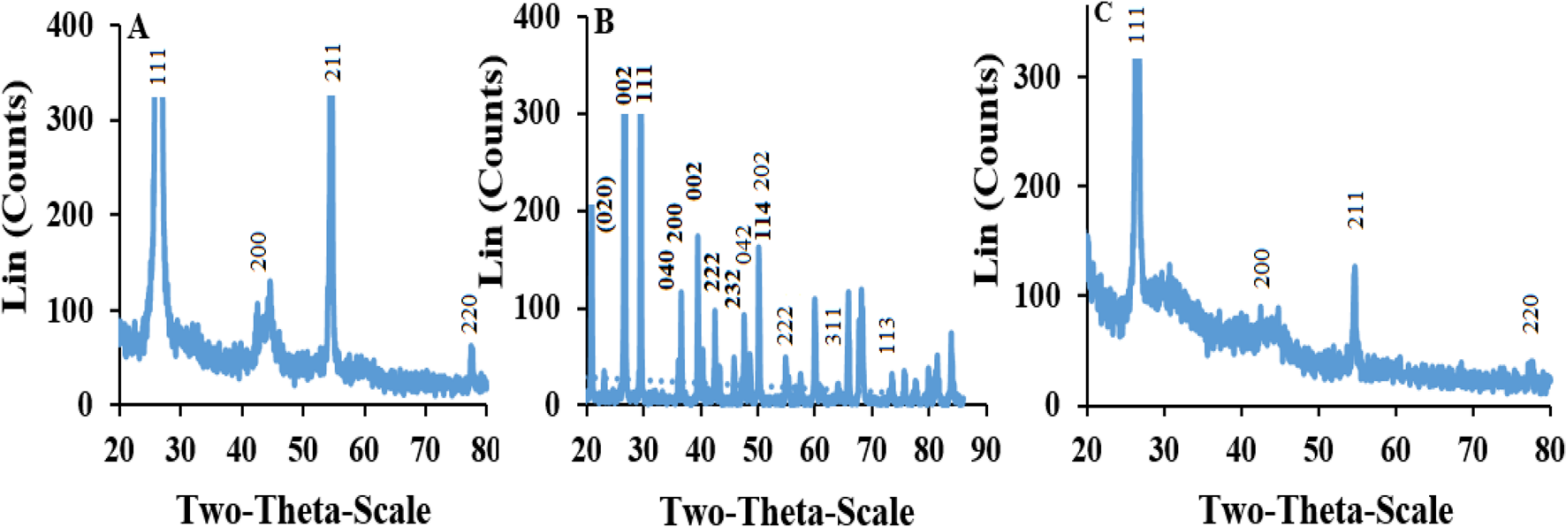
XRD patterns including (**A**) W filament, (**B**) WO_3_ generated after introduction of humidity and (**C**) W-filament after the elimination of the memory effect. Conditions: standard RH: 40–45%, applied voltages: 110 ± 1 V (AC, vs. GND), and pressure: 25–30 torr.

Formation of this reagent was evidenced via surface analyses of the tungsten filament before and after reaction with the water molecules using the driving force of the thermionic electrical current by the XRD spectrometry. The XRD patterns were shown in Fig. 3. According to the XRD pattern (Fig. 3A), the peaks positioned at different 2θ angles were related to the tungsten (Fig. 3A, C). However, probably due to the presence of coarse conditions, especially high temperature, of the reported reaction, it was likely to present some impurities on the surface. No changes were observed between the fresh W-filament and that interacted with H_2_O vapor (RH: 40–45%), in the absence of any thermionic electrical current flow. Whereas, after applying the electrical potential (i.e., 110 ± 1 V, AC, vs. GND) and visualizing the thermionic radiation, appearance of partially sharp peaks (Fig. 3B) at different angles that interpreted according to the XRD database and also via comparison with previously published articles, pointed to the formation of almost WO_3_ and WO_3_.xH_2_O with different lattices, shown in Fig. 3B, as the product of the reaction, which agreed with those reported in the literature^37,38^.

Though, comparing the XRD patterns of fresh (Fig. 3A) and memory-effect eliminated (Fig. 3C) filaments show no significant difference(s). This observation reveals the ability of this sensor for repeated experiment without hysteresis, which is an important property of humidity sensors to consider as promising candidate for commercial use.

It is also notable that, owing to some existed instrumental limitation such as hard sample introduction of the W filament with large fragility and small size; as well as, because of the presence of very thin film and probably irregularly modified compound(s) on the W support, it was impossible to directly analyze the sample with X-ray photoelectron (*XPS*) spectrometry. At this condition, we had to focus on the proposed mechanism during RH introduction > 60% only using FT-IR and XRD analyses.

According to the above discussion, the probable behavior (mechanism) of the RH sensing of the fabricated sensor can be attributed to the reaction between the tungsten filament and water vapors. Regarding the results of FT-IR spectra and XRD patterns can be concluded that, the sensing mechanism is based on the formation of almost WO_3_.xH_2_O during interaction between the H_2_O molecules and tungsten filament.

The chemical reaction between tungsten and water molecules has also been evaluated thermodynamically. Based on the chemical physics studies^39,40^, the only reactions (to the best of knowledge) between the tungsten and the water molecule were endothermic process with apparent activation energy that was estimated to be 132.7  ± 1.1 kJ mol^−1^^40^. This extent of energy can easily be provided by thermionic emission. The thermodynamic reactions are therefore as follows (Eqs. 1–3)^39,40^.

1 $${\text{W}} + 2{\text{H}}_{2} {\text{O}} \to {\text{WO}}_{2} + 2{\text{H}}_{2}$$

2 $${\text{W}} + 3{\text{H}}_{2} {\text{O}} \to {\text{WO}}_{3} + 3{\text{H}}_{2}$$

3 $${\text{WO}}_{3} + {\text{xH}}_{2} {\text{O}} \to {\text{WO}}_{3} {\text{.xH}}_{2} {\text{O}}\;({\text{x}} = 1 - 2)$$

As shown, both the thermodynamic and spectroscopic results pointed to the formation of WO_3_.xH_2_O as the electrochemical production of the H_2_O molecules and tungsten filament at the optimum condition by the driving force of the thermionic current (radiations). This theoretical evidence was therefore considered as another proofs, beside the experimental evidenced, obtained from the FT-IR and XRD analyses.

### Optimization of effective parameters

Parameters having strong influence for the humidity measurement purposes included red, green and blue components, length (resistance) of the filament, applied electrical potential, pressure and the flow rate of Ar as carrier gas. The optimization process was discussed in detail in the following subsections.

#### Selection of RGB components

**Figure 4:**
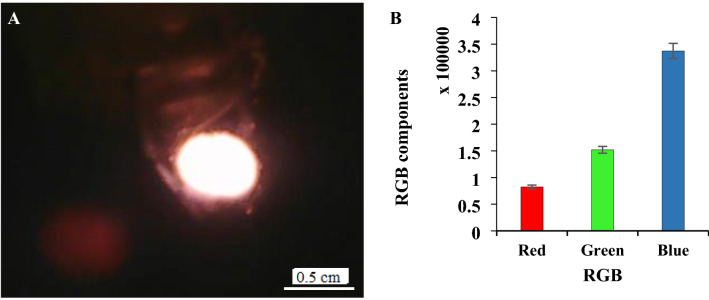
(**A**, **B**) Photographic image of thermionic emission of the W-filament and (**B**) histogram of red, green and blue components during introduction of 40–45% RH using filament with 6.00 ± 0.01 mm length at 25–30 torr pressure and applying 110 ± 1 V (AC, vs. GND). The data are the average of three independent analyses. Error bar: ± relative standard deviation.

To reach the highest sensitivity (light intensity) during reaction between tungsten and water molecules, one of the most important parameters was the selection RGB components for having maximum sensitivity and light intensity. As explained before, summation of each R, G and B components of each photographic image was estimated independently, pixel-by-pixel, using the VB_6_ program. To optimize this factor, electrical potential of 110 ± 1 V (AC, vs. GND) was applied to the tungsten filament with 6.00 ± 0.01 mm length (measured using a micrometer) at 25–30 torr pressure during introduction of standard humidity of 40–45% RH. The obtained photographic image has been shown in Fig. 4A. The summations of red, green and blue components of the thermionic radiation under above mentioned conditions has been shown in Fig. 4B.

As it is clear maximum sensitivity was observed for the blue component. Therefore, this component was selected as optical probe (analytical signal) during humidity measuring process.

#### Electrical potential

**Figure 5:**
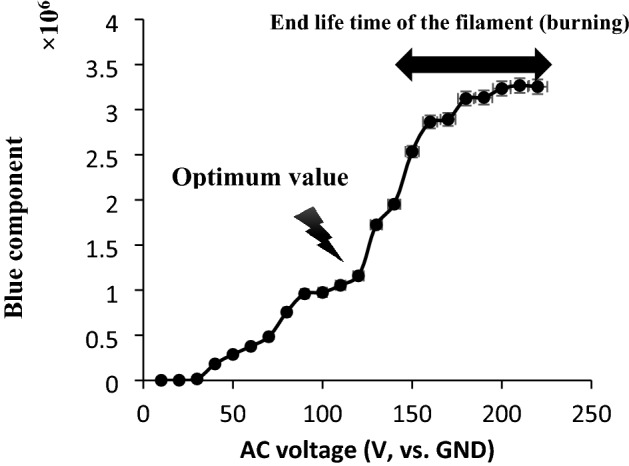
Correlation between the blue components and different applied potential. The data are the average of three independent analyses. Conditions: length of filament 6.00 ± 0.01 mm, RH: 40–45%, and temperature 40–45 °C. The data are the average of three independent analyses. Error bar: ± relative standard deviation.

To optimize the electrical potential, different electrical potentials, ranged between 10 and 220 (± 1) V (AC, vs. GND), were applied to the tungsten filament with 6.00 ± 0.01 mm length and at 25–30 torr pressure. The correlation between the blue component and the electrical potential has been shown in Fig. 5.

As shown, there are two plateaus for stable response of blue component, but, due to the flickering effect as well as small lifetime of the tungsten filament during applying high AC voltage, optimum applied potential, 110 ± 1 V, was selected from the first stable region between 90 and 115 (± 1) V (vs. GND).

#### Effect of pressure

**Figure 6:**
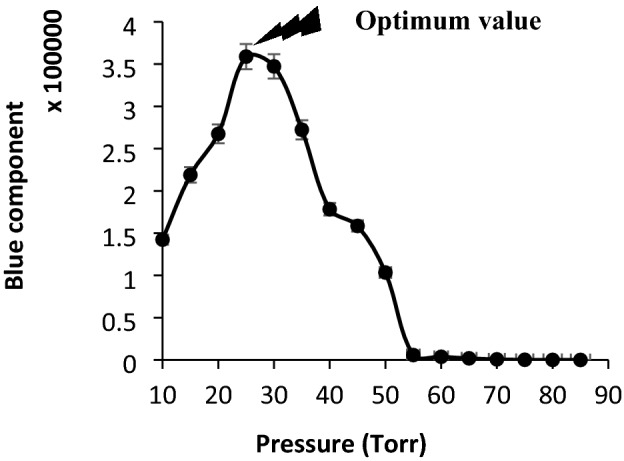
Effect of pressure on the sensitivity of the humidity optical sensor. Conditions: length of filament 6.00 ± 0.01 mm, RH: 40–45%, applied voltage: 110 ± 1 V (AC, vs. GND) and temperature 40–45 °C. The data are the average of three independent analyses. Error bar: ± relative standard deviation.

Another factor having important influence on the thermionic radiation was the pressure. To optimize this factor, the system was set during individually setting the pressure of the cell between 5 and 90 torr throughout applying 110 ± 1 V (AC, vs. GND) potential. The results have been shown in Fig. 6.

According to the results, maximum sensitivity was observed during applying vacuum condition between 25 and 30 torr. Therefore, this range was selected as the optimum pressure.

#### Effect of different lengths of tungsten filament

**Figure 7:**
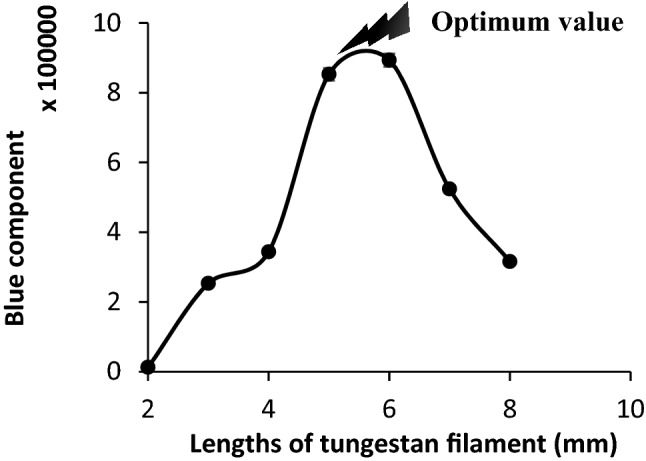
Effect of different lengths of tungsten filament during introduction of 40–45% RH. Conditions: RH: 40–45%, applied voltage: 110 ± 1 V (AC, vs. GND), pressure: 25–30 torr, and 40–45 °C temperature. The data are the average of three independent analyses. Error bar: ± relative standard deviation.

At constant electrical potential, the length of the tungsten filament directly affected the electrical resistivity and the electrical current flow through the tungsten filament. To optimize this factor, different lengths of the tungsten filament, ranged between 2.00 and 8.00 (± 0.01) mm were tested under the optimum condition. The results have been shown in Fig. 7.

Based on the results, a tungsten filament with a thickness of 6.00 ± 0.01 mm was selected as optimum length.

#### Flow rate of Ar as carrier gas

In order to prevent the tungsten filament from burning by oxygen gas in air and also from evaporating the filament, argon gas was used as carrier gas instead of air. To optimize the flow rate of the carrier gas, different flow rates of Ar gas were tested through the introduction of 40–45% RH at the optimum condition such as length of filament 6.00 ± 0.01 mm, RH: 40–45%, applied voltage: 110 ± 1 V (AC, vs. GND) at 25 °C. Based on the results, the little fluctuation was observed in the vacuum condition at flow rates larger than 5.00 ± 0.03 mL min^−1^. Therefore, this flow rate was selected.

#### Effect of DC and AC electrical potentials

Effect of type of electrical voltages (AC and DC) on analytical signal (blue component of RGB) was also investigated. The obtained results indicated that, maximum blue component was provided when AC voltage was applied, versus the DC potential (under similar conditions) at the optimum conditions. This process was almost attributed to the (i) segmented thermionic radiations during the applying the AC potential and (ii) periodic resting the tungsten figment during the AC alternates.

#### Effect of temperature

**Figure 8:**
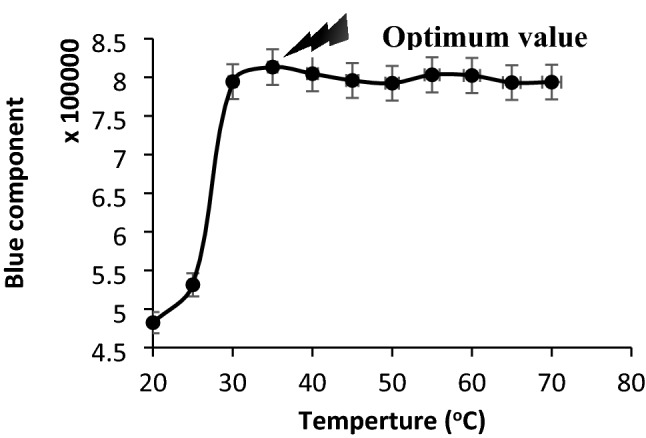
Optimum temperature for water vapors introduced to the analyzing system. Conditions: standard RH: 40–45%, applied voltages: 110 ± 1 V (AC, vs. GND), and pressure: 25–30 torr. The data are the average of three independent analyses. Error bar: ± relative standard deviation.

Linear stability of the response of the sensor was observed during providing reverse changes between RH% and temperature ranging between 20 and 70 °C for RH 40–45% (Fig. 8).

According to the results, the effect of water container temperature on the response of the fabricated sensor at different temperatures after the range of 30–35 °C was analyzed for humidity sensing purposes. As maximum sensitivity was observed at temperature between 30 and 35 °C, therefore this thermal range was selected as optimum temperature of the water vapored prior introduction to the analyzing system.

### Calibration of relative humidity and stability study

**Figure 9:**
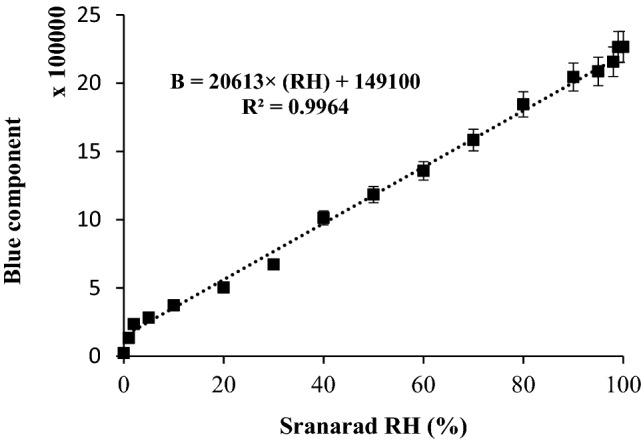
Calibration curve of RGB parameter vs. %RH. Conditions: standard RH: 0–100%, applied voltages: 110 ± 1 V (AC, vs. GND), pressure: 25–30 torr, and temperature 40–45 °C. The data are the average of three independent analyses at optimum conditions. Error bar: ± relative standard deviation.

The calibration curve of the thermionic emission-based optical sensor ranging from 2 to 98% RH has been shown in Fig. 9. Up to our knowledge, this wide range of linearity to response have not been reported for optical humidity sensors.

**Figure 10:**
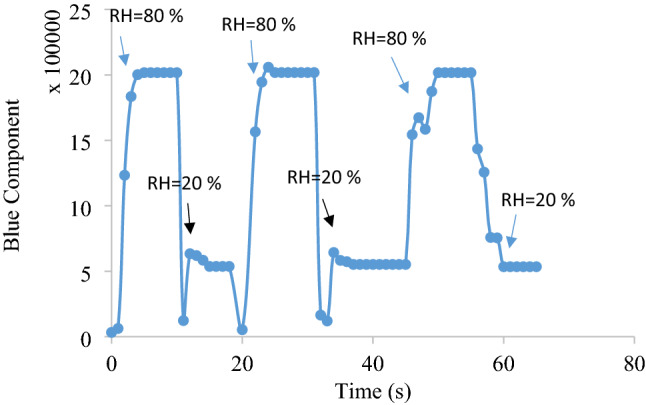
Diagram representing the hysteresis of the fabricated sensor Conditions: standard RH: 20 and 80% (Sequential introduction), applied voltages: 110 ± 1 V (AC, vs. GND), pressure: 25–30 torr, and temperature 40–45 °C. The data are the average of three independent analyses. Error bar: ± relative standard deviation.

The rate of the change in the humidity of the chamber was controlled for having enough time to stabilize the response of the sensor during sweeping the humidity. Based on 90% of maximum response time (i.e., *t*_*90*_), the response time of the fabricated RH sensor was estimated to be maximum 4.5 s. In addition, the recovery time of the sensor based on 90% of minimum response (*t*_*90*_) was found to be maximum 5.0 s. The hysteresis during rapid and alternative contacting the optical RH sensor to two sequential conditions such as 20 and 80% RH during three sequential analyses has been shown in Fig. 10.

The obtained results reveal the stability and reproducibility of the fabricated RH sensor at hard conditions. The results were also compared to that of RH reference sensor. Minimum difference (< 4.0%, n = 3) was observed between these two RH sensors that revealed the acceptable and stable behavior of the proposed RH sensor during sensing RH% at different real environments.

In this study, detection limit was defined as three folds of the standard deviation of blank to the calibration sensitivity. This value was estimated to less than 0.5% RH. Due to the hard instrumental conditions of this system, it was not possible to estimate the accurate value for the detection limit. Consequently, it was roughly estimated theoretically based on t-statistical test. More improved detection limit was evaluated for the tungsten-based humidity optical sensor, in comparison with other types of optical sensors^31–33^.

### Selectivity and interference studies

Both following experiments were conducted in other gas:H_2_O concentration ratio of about 100:1 to show the ability of proposed humidity sensor. The probable interfering effect of different foreign gaseous species was investigated in the room temperature. For this purpose, the humidity sensor was placed in the chamber and enough excess (at least 100-fold excess) of foreign gases such as CO, CO_2_, acetylene, bus and car exhaust individually introduced to the cell at RH = 40–45%. Steady state, reproducible results with no hysteresis and no significant raise and falling was observed during introducing different foreign species to the standard RH sample. Fortunately, no noticeable change in blue parameter clearly revealed the reliability of the fabricated sensor for the trustworthy humidity sensing purpose. This result was not comparable with the thermionic electrical current.

**Figure 11:**
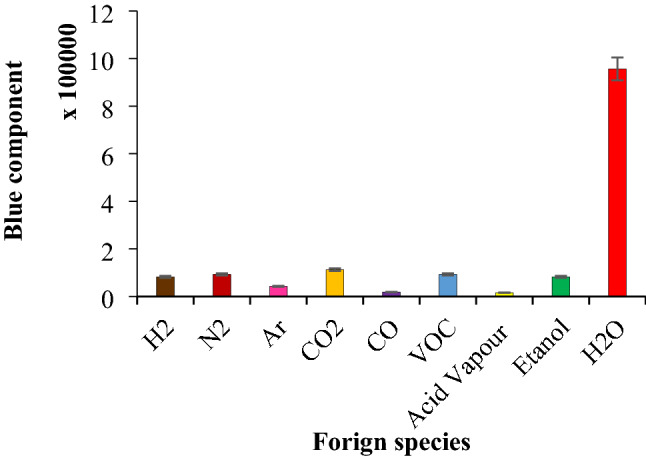
Selectivity of developed sensor. Conditions: standard RH: 40–45%, applied voltages: 110 ± 1 V (AC, vs. GND), and pressure: 25–30 torr. Tolerance ratio: at least 100-fold excess. The data are the average of three independent analyses at optimum conditions. Error bar: ± relative standard deviation.

In addition, the selectivity of proposed sensor was investigated against CO, CO_2_, N_2_, Ar, He, H_2_ and volatile organic compounds (*VOCs*), ethanol, as well as vapor of acids (HCl vapor in this work). In another word, as selectivity means that sensors does not respond to other species, that is, there was no interference of each CO, CO_2_, VOC, etc. Consequently, when the measurement was made in bus or car exhaust, the response did not changed. In fact, the sensor presented similar responses and there was no matrix effect (or matrix interference). Therefore, the results exhibited a good selectivity for developed sensor (Fig. 11).

### Reusability of the sensor

The repeatability of the fabricated humidity sensor was also evaluated at 45–50% RH. The results revealed acceptable relative standard deviation (RSD%, 4.18% (n = 5) for the fabricated optical sensor. Generally, more reproducibility of the optical imaging process for choosing as detection method in comparison with the thermionic electrical current (7.46%, n = 5) under similar conditions points out the right selection of the image processing for the RH sensing purpose.

Also, the RSD% (reproducibility) during analyses of five RH standard samples during at least five replicated analyses was estimated to be 6.05% (n = 3) pointing out to the acceptable reproducibility of the sensor for RH sensing purposes. However, pressure dependency of the desorption of this thin layer from the surface of the W filament causes to have reusable (renewability) of the RH optical sensor without any memory effect(s), which was considered as the noticeable feature of this introduced sensing device.

### Real sample analysis

The reliability of the proposed RH sensor was evaluated via determination of the RH% in different kinds of real gaseous sample, and then comparing the results with them obtained by the reference RH probe, under similar conditions. The results point out relative error percentages as maximum as ± 2.31%, which showing the applicability of this RH sensor for the analysis of different real samples.

### Comparisons

Comparison between the developed sensor and other sensors, reported in the literature for the RH sensing has been summarized in Table 1. In this comparison, two significant figures of merit: the response time and linear detection range (LDR) of the sensors were compared. Having the widest linearity of humidity sensing and acceptable short response time of the proposed method showed that, the fabricated RH sensor was considered as satisfactory humidity probe with high accuracy and precision.

**Table 1:** Comparison between the developed sensor and other RH sensing probes reported in the literature.

Sensor	LDR (% RH)	Response time (s)	Refs.
Depositing the hydrogels poly-hydroxyethyl methacrylate, poly-acrylamide, poly-N-vinyl pyrrolidinone and agarose on optical fiber in order to study their behavior with humidity	10–100	90.00	^41^
Highly porous nanostructured titanium dioxide thin film as an optical interference filter	13–71	0.27	^44^
Spin coated films of Co-Polyaniline nanocomposite for their transmission properties using He–Ne laser	20–95	8.00	^45^
A hetero-core optical fiber structure that was coated with hygroscopic polymer layers by a layer-by-layer technique, producing a [poly-glutamic acid/poly-lysine] nanostructured overlay	20–90	0.40	^42^
Microrings assembled with polyacrylamide (PAM) microfibers	5–71	0.12	^46^
An optical fiber Fabry–Perot interferometric sensors to detect humidity by depositing a hydrophilic coating material on the optical fiber tip	22–80	0.24	^43^
Thermionic emission of tungsten filament	2–98	≤ 5	This work

As expected, the linear range of RH sensors should be ideally within 0–100%. Whereas, but about most of the commercial RH sensing devices (to the best of knowledge), we do not see such an ideal condition (Table 1). This is related to the level off condition of the sensor, almost due to small improved detection limit (especially at small RH%), besides saturation of the probe with the analyte at high RH%. That is why, the scientists often report limit of quantitative and limit of linearity for the introduced sensors. Fortunately, about our RH sensor in this research, the linear range is wide enough that make it suitable for different applications.

## Conclusions

Humidity sensor was constructed based on chemical reaction between water vapor and tungsten filament. About this system, optical image of the tungsten filament was considered as an appropriate detection system for the introduced RH sensing device during the image processing using the CCD camera. Parameters having effective influence on the sensitivity of the humidity sensor include conditioning of the tungsten filament, length (resistance) of the tungsten-based filament, electrical potential, effect of vacuum, flow rate of Ar as carrier gas and the temperature. Effect of interferences of CO, CO_2_, acetylene, bus and car exhaust and selectivity of the proposed sensor against N_2_, H_2_, CO, CO_2_, Ar, He, and different VOCs show the promising results to commercially use this sensor. The application of this sensor was also evaluated via estimation of RH% in different environmental samples. Generally, it can be concluded that, the fabricated humidity optical sensor has more improved detection limit (less than 5.0% RH), higher saturated limit (> 99.0% RH), the least relative standard deviation (RSD = 4.18%), shorter rise time (less than 5 s) and also the highest linearity (R^2^ = 0.9964), compared to those estimated for other types of RH sensors. The introduced sensor therefore showed enough novelty such as (1) no significant interference of this introduced RH sensor to detect humidity against other common gaseous ones, (2) high selectivity, (3) acceptable reproducibility, (4) no serious hysteresis, (5) high sensitivity, (6) the regeneration (reusability) of the sensor’s surface for the next experiments and so on. All the results approve that the fabricated RH sensor is considered as acceptable humidity probe with high accuracy and precision.
